# Active immunoprophyilaxis with uromune® decreases the recurrence of urinary tract infections at three and six months after treatment without relevant secondary effects

**DOI:** 10.1186/s12879-019-4541-y

**Published:** 2019-10-28

**Authors:** Cristóbal Ramírez Sevilla, Esther Gómez Lanza, Juan Llopis Manzanera, Jose Antonio Romero Martín, Miguel Ángel Barranco Sanz

**Affiliations:** 1 Mataró Hospital, Barcelona, Spain; 2 Urologist Moisés Broggi Hospital, Sant Joan Despí, Barcelona, Spain

**Keywords:** Urinary tract infection, Prevention, Bacterial vaccine

## Abstract

**Background:**

To prospectively analyze the efficacy of uromune® in the prevention of uncomplicated recurrent urinary tract infections at 3 and 6 months, and according to gender and menopause.

**Methods:**

From September 2011 to December 2017 uromune® was administered sublingually every 24 h along 3 months to 784 patients with history of three or more uncomplicated urinary tract infections in the 12 months prior to the first visit.

The variables analyzed with statistical package system for science version 15.0 were age, gender, number of urinary tract infections with positive urine culture in the first consultation, and 3 and 6 months after the end of treatment.

The results with positive urine culture were registered at 3 and 6 months after the end of the treatment according to gender and also in the menopausal group with respect to pre-menopausal women.

**Results:**

Mean age was 73.5 years. 82.7% were women and 94.3% menopausal.

The number of episodes of urinary tract infections in the 12 months prior to uromune® were 3 in 37.2%, 4 in 28.1%, 5 in 19.5%, 6 in 9.6%, 7 in 4%, 8 in 1.4%, 9 in 0.1% and 10 in 0.1%.

Three months after uromune® 44.1% had 0 urinary tract infections and 27.6% had 1. After 6 months the results were 0 urinary tract infections in 32.3% and 1 in 32.4%.

Women had 0 urinary tract infections after 3 months in 45.4% and 1 in 28.5%. At 6 months the female had 0 episodes in 32.7% and 1 in 33.2%.

Menopausal women had 0 urinary tract infections at 3 months in 46.5% and 1 in 28% and at 6 months scored 0 episodes in 33.6% and 1 in 32.9%.

**Conclusions:**

Uromune® was highly effective to reduce the number of episodes of urinary tract infections at three and six months of follow-up. Uromune® reduced the number of episodes to zero or one in 71.7 and 64.7% at three and six months with minimal side effects.

The best results were observed in women over 50 years old.

Sublingual immunoprophylaxis with uromune® could be the treatment of first choice in the prevention of uncomplicated recurrent urinary tract infections according to the sample analyzed.

## Background

Urinary tract infections (UTI) are the most frequent bacterial infections [1, 2], and were the reason for more than six million consultations in the USA during the year 2007 [3]. According to Nicolle LE [2], UTI are the second cause of hospitalization due to infectious disease in people over 65 years. It is also estimated that one to three women under 24 years of age have required antibiotic treatment because of an episode of UTI. Additionally, 15% of sexually active women will have a urinary infection, and up to 60% of women will have an episode of UTI throughout life [4, 5].

Urinary tract infections can be classified according to their location in the upper or lower urinary tract. They can be acute or chronic; complicated or uncomplicated; symptomatic or asymptomatic; and initial, persistent or recurrent [1].

Uncomplicated UTI are defined as urine infections that occur in the absence of functional or anatomical alterations of the urinary tract. In contrast, complicated UTI occur secondarily to functional or anatomical urinary disorders that facilitate the persistence, recurrence, and failure of treatment [1].

Urinary tract infections are symptomatic when there are clinical urinary symptoms that indicate infection, such as micturition, dysuria, or pollakiuria; and there are pathogens verified in the urine culture [6].

Urinary tract infections are recurrent when at least two episodes appear over a 6 months period, or three over a 12 months period [7, 8].

The most prevalent location of UTI is the bladder, where they cause cystitis. Women can be up to 30 times more predisposed than men to suffer from them [5].

Among females uncomplicated UTI have a higher incidence between 18 and 39 years of age, coinciding with the period of highest sexual activity. On the other hand, with the increase in life expectancy of the population, it is estimated that UTI will rise in people over 65 years of age in the coming decades. It is also estimated that up to 25% of women will have some recurrence, and 27% will be within the same year [9].

The most frequent pathogen isolated in patients with UTI is *Escherichia coli* with 80%, followed by *Klebsiella pneumoniae*, *Proteus spp*, *Enterococcus spp,* and *Staphylococcus saprophyticus* [10].

Each episode of urinary infection in premenopausal women is associated with 6.1 days of symptoms, 2.4 days of school or work absenteeism and 0.4 days of need to be bedridden [9]. The cost of treating UTI in the US was estimated in 2008 at more than 2.5 trillion dollars^4^. For these reasons, UTI have a great impact on the quality of life and the economy [11].

The recommendation in the prophylaxis of recurrent urinary tract infections is continued antibiotic treatment, usually with sulfamethoxazole 200mgr / trimetroprim 40mgr (SMX / TMP) orally every 24 h or nitrofurantoin 100mgr orally every 24 h for 6 months [10–12]. Prolonged consumption of antibiotics is not free of side effects. They can damage the intestinal microbiota and favor the emergence of bacterial resistance.

In view of this situation and as an alternative to antibiotics for the prevention of uncomplicated recurrent UTI, a bacterial vaccine called uromune®, manufactured by Inmunotek (Madrid, Spain) and distributed by Q Pharma (Alicante, Spain) was marketed in Spain in October 2010.

## Objective

To prospectively analyze the efficacy of uromune® in the prevention of uncomplicated recurrent UTI 3 and 6 months after the end of treatment. To analyze the distribution of positive urine cultures post-treatment with uromune® according to gender and in menopause with respect to pre-menopause.

## Methods

This study was prospective, descriptive and not comparative. The inclusion of patients was performed consecutively in an Excel database.

During a 74-month period, from September 2011 to December 2017, uromune® was administered to a total of 784 patients from two hospital centers, all with a history of three or more uncomplicated UTI in the 12 months prior to their first visit. Six hundred nineteen patients were from Mataró Hospital and 165 from Moisés Broggi Hospital in Sant Joan Despí, both centers belonging to the province of Barcelona, Spain.

Uromune® was administered sublingually in the form of a spray, with two pumps following fasting and at a temperature not higher than 24 degrees Celsius, every 24 h for a continuous period of 3 months. The treatment was only interrupted in those episodes of a fever over 38 degrees Celsius of any origin, restarting it once the febrile episode was resolved.

Each pump of uromune® was equivalent to a suspension of 10 to the 9 inactive whole bacteria / ml, with an equal percentage for the four strains of the four most common pathogens causing UTI in Spain: *Escherichia coli, Klebsiella pneumoniae, Proteus vulgaris and Enterococcus faecalis.* The content was left under the tongue for 1 min, without the patient swallowing saliva for better absorption [8].

All patients initially underwent detailed anamnesis and an abdominal and genital physical examination blood analysis, urine culture and reno-vesical ultrasound in women and reno-vesico-prostatic ultrasound in men were performed. In cases of suspected complicated UTI, a urethrocystoscopy, urinary cytology, abdominal CT, uroflowmetry and urodynamics were also performed.

We defined causes of exclusion of the study the presence of neurogenic bladder, symptomatic urinary lithiasis, ureteral catheter or nephrostomy, moderate-severe urinary incontinence defined as the presence of three or more one-hour pad test equal to or greater than 50 cc, benign hyperplasia of prostate (BPH) in progression defined as patients with IPSS greater than 15 despite medical treatment combined with alpha-blockers and inhibitors of 5-phosphodiesterase, BPH and cystocele with postvoid residual greater than 100 ml and patients with Bricker urinary diversion, neobladder and cutaneous urostomy.

The variables analyzed were age, gender, and the number of UTI with positive urine culture in the first consultation and 3 and 6 months after the end of treatment.

The results were compared with positive urine culture at 3 and 6 months after the end of the treatment, according to gender and also in the menopausal group, with respect to pre-menopausal women. The pre-menopause was defined in this study as women under 50 years of age, and menopause as women of age equal to or greater than 50 years old.

It is a prospective study started in September 2011 and six urologists have participated. All the patients came from urban areas and were selected consecutively until December of 2017, taking into account that all had had three or more episodes of uncomplicated UTI in the 12 months prior to the start of treatment with uromune®, and taking into account as well the exclusion criteria mentioned before.

The variables studied were recorded in an Excel database and subsequently exported to carry out the process of the data to the SPSS system (Statistical Package for the Social Sciences) version 15.0, which is a statistical analysis software through syntax and processes.

From the sample of the study, the metric data was obtained for the quantitative variables that allowed the calculation of the statistical indexes, and the non-metric data for the categorical variables.

The qualitative variables were measured as number and proportion. The quantitative variables were expressed by the mean and median central tendency statistics, and by the statistics of standard deviation and range dispersion. To compare proportions, the Chi-square test was used with the Fisher modification when necessary. The comparison between quantitative variables was performed using the Student’s t test.

In all patients included in the sample of this study, uromune® was used as the first choice in the prophylaxis of uncomplicated recurrent UTI. We defined a favorable or effective response to the existence of zero or one episode of UTI at 3 and 6 months after finishing treatment with uromune®.

The side effects of uromune® therapy were local and rare. There were registered 8 patients with dry mouth, 4 with gastritis and 3 with sickness. The duration was less than 7 days except in 2 cases of dry mouth that lasted 15 days. Three patients discontinued the treatment for 10 days due to fever secondary a respiratory infection and subsequently continued the therapy. No secondary effects were grounds for abandonment of treatment.

## Results

The mean age was 73.5 years, with a range of 19–97 years and a standard derivation of 12.75. 82.7% (648) were women and 17.3% (136) men. 94.3% (611 women) were menopausal and 5.7% (37) pre-menopausal. The most frequently isolated bacteria was *Escherichia coli* followed by *Klebsiella pneumoniae*. Three hundred eighty patients (48,5%) presented *Escherichia coli* and 273 (34,8%) presented *Klebsiella penumoniae.*

**Fig. 1:**
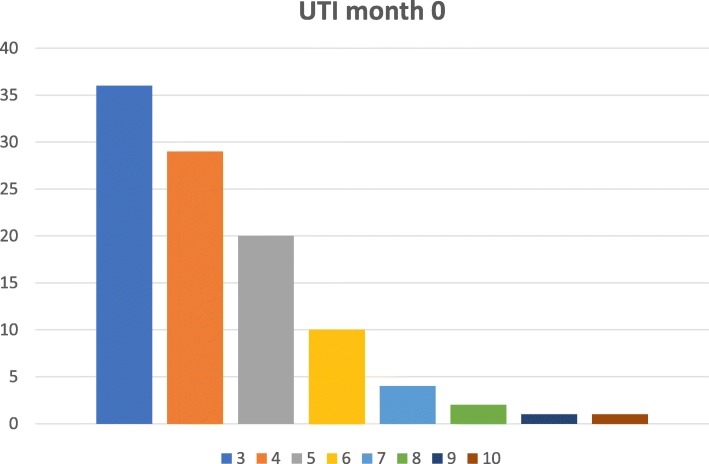
Distribution of the UTI number in the first visit

The number of episodes of UTI with positive urine culture in the 12 months prior to the start of treatment with uromune® was 3 in 37.2% (292 patients), 4 in 28.1% (220), 5 in 19.5% (153), 6 in 9.6% (75), 7 in 4% (31), 8 in 1.4% (11), 9 in 0.1% (1) and 10 in 0.1% (1) (Fig. 1).

**Fig. 2:**
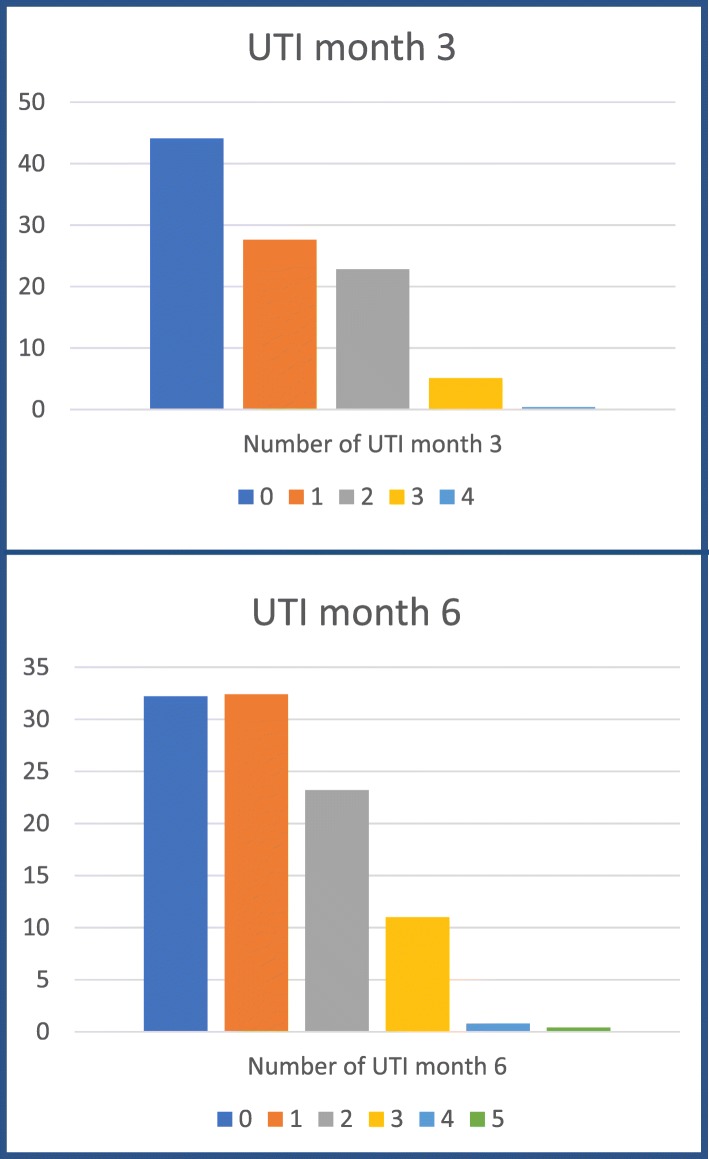
UTI results three and six months after treatment with uromune®

Three months after finishing treatment with uromune, 346 patients (44.1%) had 0 UTI, 216 (27.6%) had 1 UTI, 179 (22.8%) had 2 UTI, 40 (5.1%) had 3 UTI, and 3 (0.4%) had 4 UTI. After 6 months of treatment with uromune® the results were: 0 UTI in 253 patients (32.3%), 1 UTI in 254 (32.4%), 2 UTI in 182 (23.2%), 3 UTI in 86 (11%), 4 UTI in 6 (0.8%), and 5 UTI in 3 (0.4%) (Fig. 2).

Three months after the treatment, uromune® reduced the number of UTI episodes by 0 or 1 in 71,7% of patients, making it an effective response to treatment. At 6 months there was a reduction to 0 or 1 episodes in 64.7%.

**Fig. 3:**
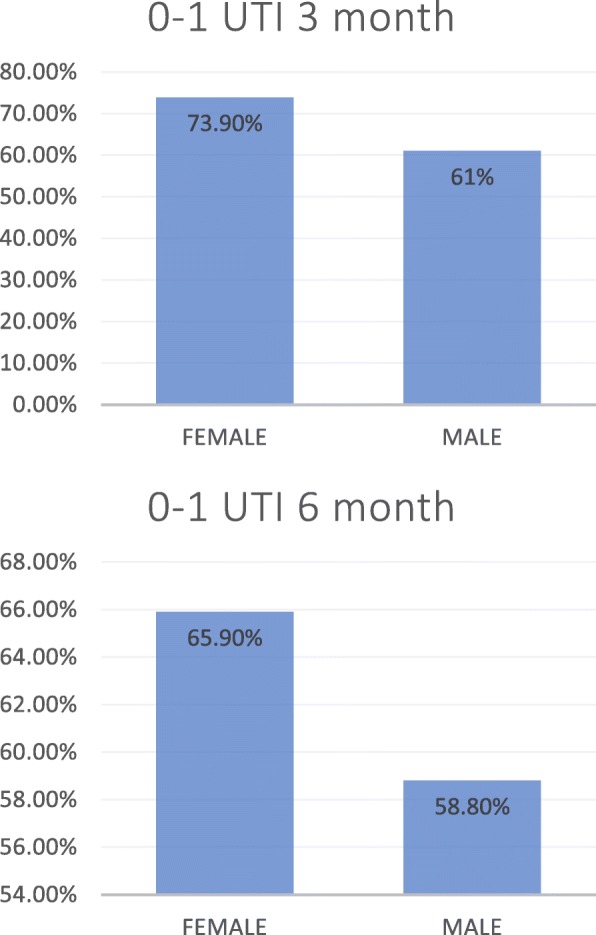
Results with zero or one UTI according to sex

According to gender, women had 0 UTI after 3 months of ending uromune® in 45.4% (294), 1 UTI in 28.5% (185), 2 UTI in 21.3% (138), 3 UTI in 4.3% (28) and 4 UTI in 0.5% (3). Among males, the response at 3 months was 0 episodes in 38.2% (52), 1 UTI in 22.8% (31), 2 UTI in 30.2% (41), and 3 UTI in 8.8% (12). At 6 months, females had 0 episodes in 32.7% (212), 1 UTI in 33.2% (215), 2 UTI in 23.3% (151), 3 UTI in 9.8% (64), and 4 or 5 UTI in 0.5% (3). Males presented at 6 months 0 episodes in 30.1% (41), 1 UTI in 28.7% (39), 2 UTI in 22.8% (31), 3 UTI in 16.2% (22), and 4 ITU in 2.2 (3) (Fig. 3).

**Fig. 4:**
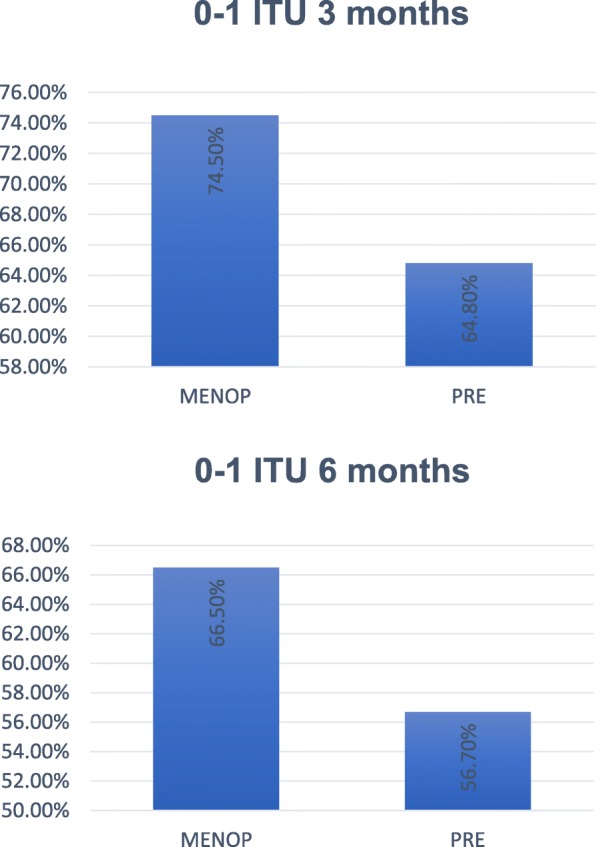
Results with zero or one UTI according to menopause

Menopausal women had 0 ITU at 3 months in 46.5% (284), 1 UTI in 28% (171), 2 UTI in 20.8% (127), 3 UTI in 4.3% (26), and 4 UTI in 0.5% (3). In pre-menopause, the results were: 0 episodes in 27% (10), 1 UTI in 37.8% (14), 2 UTI in 29.7% (11), and 3 UTI in 5.4% (2). At 6 months, menopausal women scored 0 episodes in 33.6% (205), 1 UTI in 32.9% (201), 2 UTI in 23.1% (141), 3 UTI in 9.8% (60), 4 UTI in 0.2% (1), and 5 UTI in 0.5% (3). In the pre-menopausal group, the results were: 0 recurrences in 18.9% (7), 1 UTI in 37.8% (14), 2 UTI in 27% (10), 3 UTI in 10.8% (4), and 4 UTI in 5.4% (2) (Fig. 4). The results can be cheked at the Additional file 1.

## Discussion

In 1986 Frey et al. prepared the first double-blind study on the efficacy of *Escherichia coli* extract in capsule form, taken orally for 3 months for the prevention of recurrent UTI. In a group of 27 patients, it showed a decrease in dysuria, bacteriuria, leukocyturia, and the need to take antibiotics compared to placebo [13].

Later, Tammenn et al. with a similar study observed a decrease in 12 episodes of UTI in the group treated with *Escherichia coli* extract (61 patients) compared to 41 in the placebo group (59 patients) at 3 months after treatment [14].

Two other studies in 1993 and 1994 reported by Schulman CC and Magasi P showed similar results regarding the reduction in the number of UTI, and the consumption of antibiotics in patients treated with bacterial *Escherichia coli* extract versus placebo [15, 16].

In 2005 Bauer et al. published a study comparing 231 patients treated with *Escherichia coli* extract versus a placebo group of 222, demonstrating at 12 months of follow-up that the first group had 14.7% less UTI than the placebo group [17].

The study by Lorenzo-Gómez et al. compared 159 women treated for 3 months with uromune® versus 160 women who followed daily treatment for 6 months with trimetroprim / sulfamethoxazole 40/200 mg orally. The group treated with uromune® experienced a significant reduction in UTI 3 months after finishing treatment compared to the group treated with antibiotics, and this improvement was maintained at nine and 15 months of follow-up [11]. Three years later in 2015, the same authors corroborated these results with a larger sample of 360 women treated with the vaccine compared to 339 treated with the same antibiotic for 6 months, and they concluded that with the vaccine it is possible to reduce the consumption of antibiotics and thus not increase the bacterial resistance [6].

In our study with a large sample of 784 patients, the reduction of the number of UTI episodes to zero or one was observed in 71.7% of the patients treated with uromune® at 3 months, a percentage that remained at 64,7% at 6 months. These results are similar to those published by Lorenzo-Gómez et al. in 2015 with a 75% favorable response at 3 months with the vaccine [16]. On the other hand, they are inferior to 78% of zero ITU at 3 months after finishing uromune® published in February 2018 by Yang et al. although with a smaller sample of only 75 patients [18].

In the most recent study published to date, in October 2018, by Sihrar et al. [19], the results were superior to our study with 90.3% of a total of 360 patients with only up to one UTI 12 months after the end of the treatment with uromune®.

The selection of a comparative group of patients, who had received antibiotic prophylaxis for uncomplicated recurrent UTI for 6 months, to be compared with respect to the group of patients of our study, who followed uromune®, was rejected due to three reasons: the great difficulty in obtaining data in the medical records because before 2010 the medical records were not computerized in our hospital, the great variability in the data collection in terms of the antibiotic used as prophylaxis, and the fact that in many patients we could not obtain the results of the uroculture because the results had originated in different health centers of Primary Care.

## Conclusion

Uromune® was highly effective to reduce the number of episodes of urinary tract infections at three and 6 months of follow-up. Uromune® reduced the number of episodes to zero or one in 71.7 and 64.7% at 3 and 6 months with minimal side effects.

The best results were observed in women over 50 years old.

## Recommendation

Sublingual immunoprophylaxis with uromune® could be the treatment of first choice in the prevention of uncomplicated recurrent urinary tract infections according to the sample analyzed.

## Supplementary information

**Additional file 1.** Results of the statistical analysis.

## Data Availability

The datasets generated and analyzed during the current study may be publicly available but the personal data of the patients included in the study are confidential. To access the raw data, no administrative permission is required. The corresponding author, Cristóbal Ramírez Sevilla, should be contacted if someone wants to request the data by the e-mail cjrs70@yahoo.com.

## Abbreviations

BPH: Benign prostate hyperplasia
cc: Cubic centimeters
CT: Computerized tomography
IPSS: International system prostate score
mgr: Miligrams
ml: Milliliter
SMX: Sulfamethoxazole
Spp: Species
SPSS: Statistical package for the social science
TMP: Trimetropim
US: United States
UTI: Urinary tract infection

## References

[1] Foxman B (2002). Epidemiology of urinary tract infections: incidence, morbidity and economic costs. Am J Med.

[2] Nicolle LE (2005). Managing recurrent urinary tract infection in women. Womens Health (Lond).

[3] Artero A, Inglada L, Gómez-Belda A, Capdevila JA, Diez LF, Arca A (2018). The clinical impact of bacteremia on outcomes in patients with pyelonephritis or urinary sepsis: a prospective multicenter study. PLoSOne.

[4] Rahn DD (2008). Urinary tract infections: contemporary management. Urol Nurs.

[5] Naber KG, Cho Y, Matsumoto T, Schaeffer AJ (2009). Immunoactive prophylaxis of recurrent urinary tract infections: a meta-analysis. Int J Antimicrob Agents.

[6] Lorenzo-Gómez MF, Padilla-Fernández B, García-Cenador MB, Virseda-Rodríguez ÁJ, Martín-García I, Sánchez-Escudero A, et al. Comparison of sublingual therapeutic vaccine with antibiotics for the prophylaxis of recurrent tract infections. Front Cell Infect Microbiol. 2015;5:51–8. 10.3389/fcimb.2015.00050 PMC4452880 26090341

[7] Grabe M, Bishop M, Bjerklund-Johansen T, Botto H, Cek M, Lobel B et al. Guidelines on urological infections. Eur Assoc Urol. Website: http://www.uroweb.org/fileadmin/tx_aeuguidelines/2009/Full/Urological_Infections.pdf2009;Updated2009.

[8] Prieto L, Esteban M, Salinas J, Adot JM, Arlandis S, Peri L (2015). Consensus document of the Spanish Urological Association on the management of uncomplicated recurrent urinary tract infections. Working group for the recommendations in the diagnosis and management of uncomplicated urinary tract infections. Carried out under the auspices of the Spanish Association of Urology 2013. Actas Urol Esp.

[9] Hooton TM, Scholes D, Hughes JP, Winter C, Roberts PL, Stapleton AE (1996). A prospective study of risk factors for symptomatic urinary tract infection in young women. N Engl J Med.

[10] Benito-Villalvilla C, Cirauqui C, Diez-Rivero CM, Casanovas M, Subiza JL, Palomares O (2017). MV140, a sublingual polyvalent bacterial preparation to treat recurrent urinary tract infections, human dendritic cells for generating Th1, Th17 and IL- 10 responses via Syk and MyD88. Mucosal Immunol.

[11] Lorenzo-Gómez MF, Padilla-Fernández B, García-Criado FJ, Mirón-Canelo JA, Gil-Vicente A, Nieto-Huertos A (2013). Evaluation of a therapeutic vaccine for the prevention of recurrent urinary tract infections versus prophylactic treatment with antibiotics. Int Urogynecol J.

[12] Andreu A, Planells I (2008). Spanish cooperative group for the study of antimicrobial sensitivity of urological pathogens. Med Clin.

[13] Frey C, Obolensky W, Wyss H (1986). Treatment of recurrent urinary tract infections: efficacy of an orally administered biological response modifier. Urol Int.

[14] Tammen H (1990). Immunobiotherapy with Uro-Vaxom in recurrent urinary tract infection. The German urinary tract infection study group. Br J Urol.

[15] Schulman CC, Corbusier A, Michiels H, Taenzer HJ (1993). Oral immunotherapy of recurrent urinary tract infections: a double-blind placebo-controlled multicentre study. J Urol.

[16] Magasi P, Panovics J, Illes A, Nagy M (1994). Uro-Vaxom and the management of recurrent urinary tract infection in adults: a randomized multicenter double-blind trial. Eur Urol.

[17] Bauer HW, Alloussi S, Egger G, Blumlein HM, Cozma G, Schulman CC (2005). A long-term, multicenter, double-blind study of an Escherichia coli extract (OM-89) in female patients with recurrent urinary tract infections. Eur Urol.

[18] Yang B, Foley S (2018). First experience in the UK of treating women with recurrent urinary tract infections with the bacterial vaccine Uromune. BJU Int.

[19] Sihra N, Goodman A, Zakri R, Sahai A, Malde S. Nonantibiotic prevention and management of recurrent urinary tract infection. Nat Rev Urol. 2018. 10.1038/s41585-018-0106. 10.1038/s41585-018-0106-x 30361493

