# Supplementary material for: Active immunoprophyilaxis with uromune® decreases the recurrence of urinary tract infections at three and six months after treatment without relevant secondary effects
Source: BMC Infect Dis. 2019 Oct 28;19:901. doi: 10.1186/s12879-019-4541-y (PMC6819445; doi:10.1186/s12879-019-4541-y)
Supplement: Supplementary file 1 — Additional file 1. Results of the statistical analysis. [file 12879_2019_4541_MOESM1_ESM.pdf]

```
COMPUTE EDAD=(CTIME.DAYS(FechaPrescripciónVacuna-FechaNacimiento)/365.25).
EXECUTE.

DESCRIPTIVES VARIABLES=EDAD
  /STATISTICS=MEAN STDDEV RANGE MIN MAX.
```

Descriptives

Notes

Output Created		14-SEP-2019 23:15:28
Input	Data	C:\Users\mm\Documents\CONGRESOS 2017 Treballs\EUROPEO LONDON 2017 VACUNAS UROMUNE\VACUNAS_Base Dades_BMC.sav
	Active Dataset	DataSet2
	Filter	<none>
	Weight	<none>
	Split File	<none>
	N of Rows in Working Data File	784
Missing Value Handling	Definition of Missing	User defined missing values are treated as missing.
	Cases Used	All non-missing data are used.
Syntax		DESCRIPTIVES VARIABLES=EDAD /STATISTICS=MEAN STDDEV ...
Resources	Processor Time	00:00:00,02
	Elapsed Time	00:00:00,01

```
[DataSet2] C:\Users\mm\Documents\CONGRESOS 2017 Treballs\EUROPEO LONDON 201
7 VACUNAS UROMUNE\VACUNAS_Base Dades_BMC.sav
```

Descriptive Statistics

	N	Range	Minimum	Maximum	Mean	Std. Deviation
EDAD	784	78,11	19,23	97,33	73,5050	12,75228
Valid N (listwise)	784					

```
COMPUTE EDAD50a=EDAD > 50.00.
EXECUTE.

FREQUENCIES VARIABLES=EDAD50a
```

```
/STATISTICS=RANGE MINIMUM MAXIMUM MEDIAN
/ORDER=ANALYSIS.
```

Frequencies

Notes

Output Created		14-SEP-2019 23:15:28
Input	Data	C: \Users\mm\Documents\CONGRESOS 2017 Treballs\EUROPEO LONDON 2017 VACUNAS UROMUNE\VACUNAS_Base Dades_BMC.sav
	Active Dataset	DataSet2
	Filter	<none>
	Weight	<none>
	Split File	<none>
	N of Rows in Working Data File	784
Missing Value Handling	Definition of Missing	User-defined missing values are treated as missing.
	Cases Used	Statistics are based on all cases with valid data.
Syntax		FREQUENCIES VARIABLES=EDAD50a /STATISTICS=RANGE MINIMUM MAXIMUM MEDIAN /ORDER=ANALYSIS.
Resources	Processor Time	00:00:00,02
	Elapsed Time	00:00:00,02

Statistics

EDAD50a

N	Valid	784
	Missing	0
Median		1,00
Range		1
Minimum		0
Maximum		1

EDAD50a

		Frequency	Percent	Valid Percent	Cumulative Percent
Valid	Edad inferior a 50 años	41	5,2	5,2	5,2
	Edad igual o superior a 50 años	743	94,8	94,8	100,0
	Total	784	100,0	100,0	

```
FREQUENCIES VARIABLES=BACTERIA
```

Frequencies

Notes

Output Created		14-SEP-2019 23:15:28
Input	Data	C:<br>\Users\mm\Documents\CONGRESOS 2017 Treballs\EUROPEO LONDON 2017 VACUNAS UROMUNE\VACUNAS_Base Dades_BMC.sav
	Active Dataset	DataSet2
	Filter	<none>
	Weight	<none>
	Split File	<none>
	N of Rows in Working Data File	784
Missing Value Handling	Definition of Missing	User-defined missing values are treated as missing.
	Cases Used	Statistics are based on all cases with valid data.
Syntax		FREQUENCIES VARIABLES=BACTERIA<br>/ORDER=ANALYSIS.
Resources	Processor Time	00:00:00,02
	Elapsed Time	00:00:00,02

Statistics

BACTERIA

N	Valid	784
	Missing	0

BACTERIA

		Frequency	Percent	Valid Percent	Cumulative Percent
Valid	E.Coli	380	48,5	48,5	48,5
	Klebsiella pn	273	34,8	34,8	83,3
	Proteus vulgaris	81	10,3	10,3	93,6
	E. faecalis	50	6,4	6,4	100,0
	Total	784	100,0	100,0	

```
CROSSTABS
  /TABLES=Sexo BY EDAD50a
  /FORMAT=AVALUE TABLES
  /CELLS=COUNT ROW COLUMN
  /COUNT ROUND CELL.
```

Crosstabs

Notes

Output Created		14-SEP-2019 23:15:28
Input	Data	C: \Users\mm\Documents\CONGRESOS 2017 Treballs\EUROPEO LONDON 2017 VACUNAS UROMUNE\VACUNAS_Base Dades_BMC.sav
	Active Dataset	DataSet2
	Filter	<none>
	Weight	<none>
	Split File	<none>
	N of Rows in Working Data File	784
Missing Value Handling	Definition of Missing	User-defined missing values are treated as missing.
	Cases Used	Statistics for each table are based on all the cases with valid data in the specified range(s) for all variables in each table.
Syntax		CROSSTABS<br>/TABLES=Sexo BY EDAD50a<br>/FORMAT=AVALUE TABLES<br>/CELLS=COUNT ROW COLUMN<br>/COUNT ROUND CELL.
Resources	Processor Time	00:00:00,02
	Elapsed Time	00:00:00,13
	Dimensions Requested	2
	Cells Available	349496

Case Processing Summary

	Cases					
	Valid		Missing		Total	
	N	Percent	N	Percent	N	Percent
Sexo * EDAD50a	784	100,0%	0	0,0%	784	100,0%

Sexo * EDAD50a Crosstabulation

			EDAD50a		Total
			Edad inferior a 50 años	Edad igual o superior a 50 años	
Sexo	Mujer	Count	37	611	648
		% within Sexo	5,7%	94,3%	100,0%
		% within EDAD50a	90,2%	82,2%	82,7%
	Hombre	Count	4	132	136
		% within Sexo	2,9%	97,1%	100,0%
		% within EDAD50a	9,8%	17,8%	17,3%
Total		Count	41	743	784
		% within Sexo	5,2%	94,8%	100,0%
		% within EDAD50a	100,0%	100,0%	100,0%

```
 FREQUENCIES VARIABLES=ITUmes0 ITUmes3 ITUmes6
/BARCHART PERCENT
  /ORDER=ANALYSIS.
```

Frequencies

Notes

Output Created		14-SEP-2019 23:15:28
Input	Data	C:<br>\Users\mm\Documents\CONGRESOS 2017 Treballs\EUROPEO LONDON 2017 VACUNAS UROMUNE\VACUNAS_Base Dades_BMC.sav
	Active Dataset	DataSet2
	Filter	<none>
	Weight	<none>
	Split File	<none>
	N of Rows in Working Data File	784
Missing Value Handling	Definition of Missing	User-defined missing values are treated as missing.
	Cases Used	Statistics are based on all cases with valid data.
Syntax		FREQUENCIES VARIABLES=ITUmes0 ITUmes3 ITUmes6<br>/BARCHART PERCENT<br>/ORDER=ANALYSIS.
Resources	Processor Time	00:00:05,25
	Elapsed Time	00:00:02,43

Statistics

		ITUmes0	ITUmes3	ITUmes6
N	Valid	784	784	784
	Missing	0	0	0

Frequency Table

ITUmes0

		Frequency	Percent	Valid Percent	Cumulative Percent
Valid	3	292	37,2	37,2	37,2
	4	220	28,1	28,1	65,3
	5	153	19,5	19,5	84,8
	6	75	9,6	9,6	94,4
	7	31	4,0	4,0	98,3
	8	11	1,4	1,4	99,7
	9	1	,1	,1	99,9
	10	1	,1	,1	100,0
	Total	784	100,0	100,0	

ITUmes3

		Frequency	Percent	Valid Percent	Cumulative Percent
Valid	0	346	44,1	44,1	44,1
	1	216	27,6	27,6	71,7
	2	179	22,8	22,8	94,5
	3	40	5,1	5,1	99,6
	4	3	,4	,4	100,0
	Total	784	100,0	100,0	

ITUmes6

		Frequency	Percent	Valid Percent	Cumulative Percent
Valid	0	253	32,3	32,3	32,3
	1	254	32,4	32,4	64,7
	2	182	23,2	23,2	87,9
	3	86	11,0	11,0	98,9
	4	6	,8	,8	99,6
	5	3	,4	,4	100,0
	Total	784	100,0	100,0	

Bar Chart

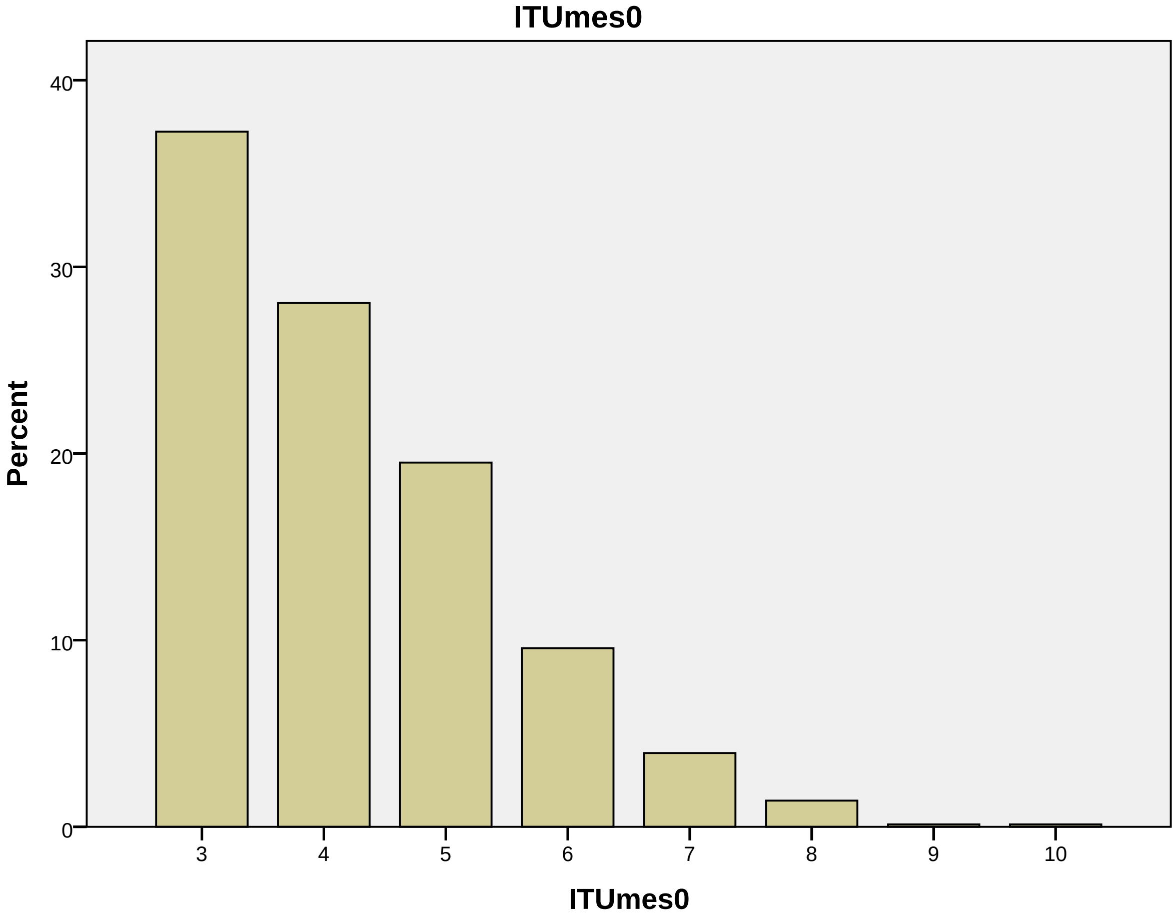
ITUmes0
Percent
40
30
20
10
0
3
4
5
6
7
8
9
10
ITUmes0

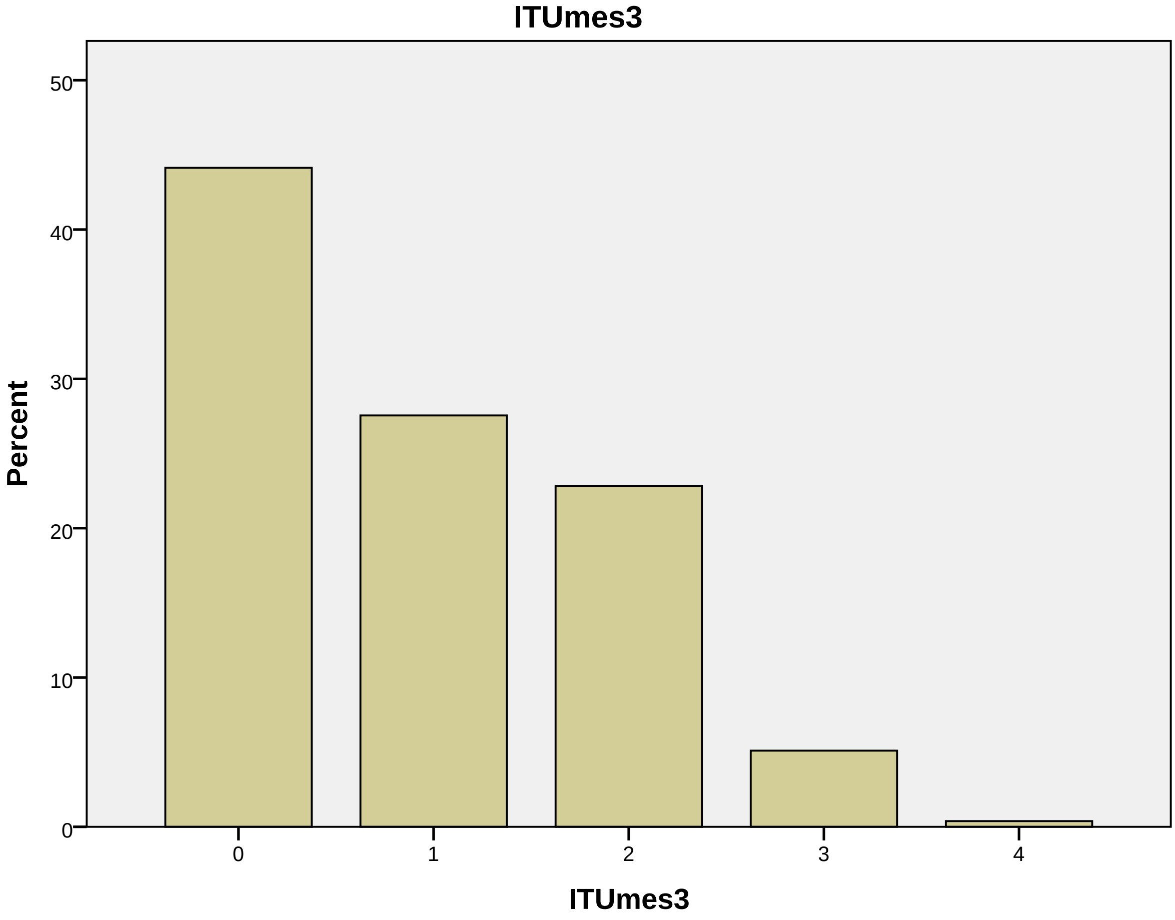
ITUmes3
Percent
50
40
30
20
10
0
0
1
2
3
4
ITUmes3

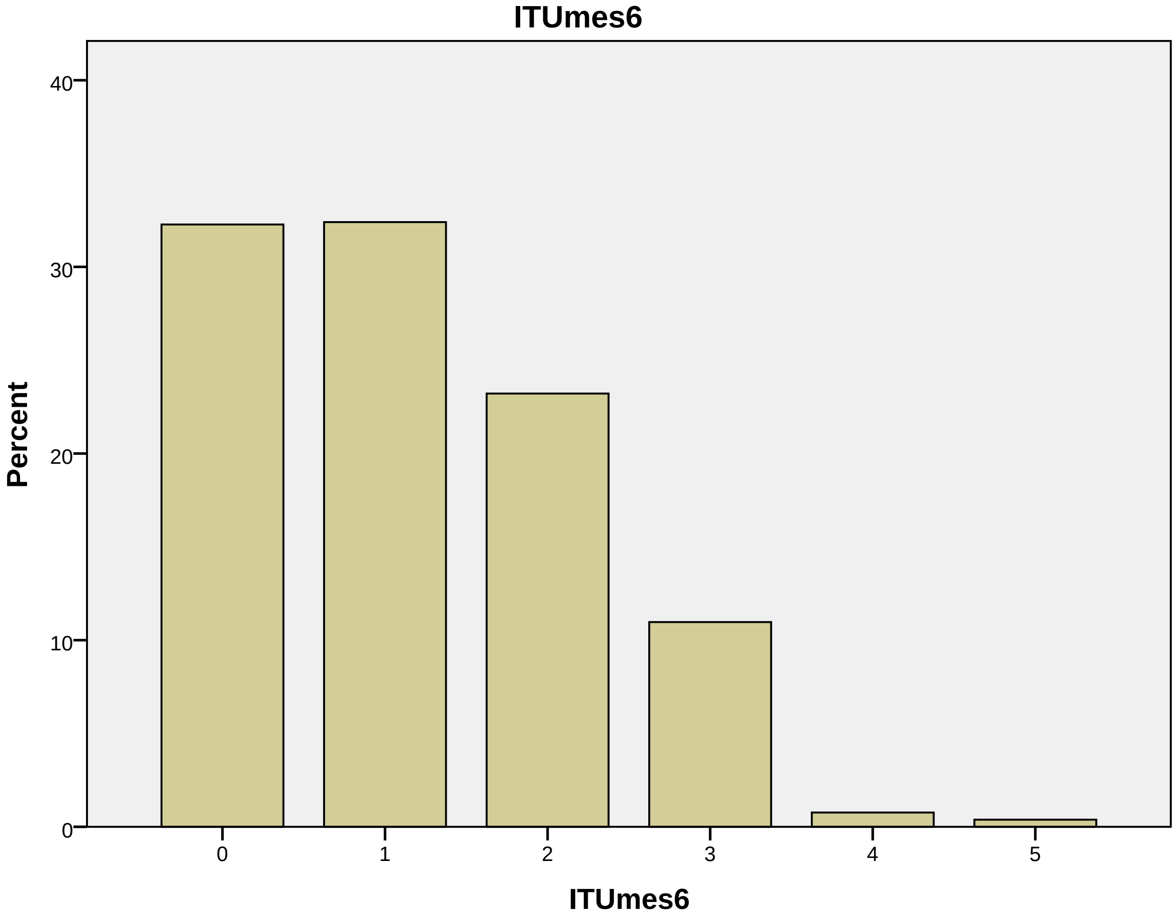


```
CROSSTABS
 /TABLES=Sexo BY ITUmes0
 /FORMAT=AVALUE TABLES
 /STATISTICS=CHISQ
 /CELLS=COUNT ROW
 /COUNT ROUND CELL.
```

Crosstabs

Notes

Output Created		14-SEP-2019 23:15:31
Input	Data	C:\Users\mm\Documents\CONGRESOS 2017 Treballs\EUROPEO LONDON 2017 VACUNAS UROMUNE\VACUNAS_Base Dades_BMC.sav
	Active Dataset	DataSet2
	Filter	<none>
	Weight	<none>
	Split File	<none>
	N of Rows in Working Data File	784
Missing Value Handling	Definition of Missing	User-defined missing values are treated as missing.
	Cases Used	Statistics for each table are based on all the cases with valid data in the specified range(s) for all variables in each table.
Syntax		CROSSTABS /TABLES=Sexo BY ITUmes0 /FORMAT=AVALUE TABLES /STATISTICS=CHISQ /CELLS=COUNT ROW /COUNT ROUND CELL.
Resources	Processor Time	00:00:00,13
	Elapsed Time	00:00:00,07
	Dimensions Requested	2
	Cells Available	349496

Case Processing Summary

	Cases					
	Valid		Missing		Total	
	N	Percent	N	Percent	N	Percent
Sexo * ITUmes0	784	100,0%	0	0,0%	784	100,0%

Sexo * ITUmes0 Crosstabulation

			ITUmes0					
			3	4	5	6	7	8
Sexo	Mujer	Count	241	182	126	62	25	10
		% within Sexo	37,2%	28,1%	19,4%	9,6%	3,9%	1,5%
	Hombre	Count	51	38	27	13	6	1
		% within Sexo	37,5%	27,9%	19,9%	9,6%	4,4%	0,7%
Total		Count	292	220	153	75	31	11
		% within Sexo	37,2%	28,1%	19,5%	9,6%	4,0%	1,4%

Sexo * ITUmes0 Crosstabulation

			ITUmes0		
			9	10	Total
Sexo	Mujer	Count	1	1	648
		% within Sexo	0,2%	0,2%	100,0%
	Hombre	Count	0	0	136
		% within Sexo	0,0%	0,0%	100,0%
Total		Count	1	1	784
		% within Sexo	0,1%	0,1%	100,0%

Chi-Square Tests

	Value	df	Asymptotic Significance (2-sided)
Pearson Chi-Square	1,043[a]	7	,994
Likelihood Ratio	1,472	7	,983
Linear-by-Linear Association	,073	1	,787
N of Valid Cases	784		

a. 5 cells (31,3%) have expected count less than 5. The minimum expected count is ,17.

```
CROSSTABS
 /TABLES=Sexo BY ITUmes3
 /FORMAT=AVALUE TABLES
 /STATISTICS=CHISQ
 /CELLS=COUNT  ROW
 /COUNT ROUND CELL.
```

Crosstabs

Notes

Output Created		14-SEP-2019 23:15:31
Input	Data	C: \Users\mm\Documents\CONGRESOS 2017 Treballs\EUROPEO LONDON 2017 VACUNAS UROMUNE\VACUNAS_Base Dades_BMC.sav
	Active Dataset	DataSet2
	Filter	<none>
	Weight	<none>
	Split File	<none>
	N of Rows in Working Data File	784
Missing Value Handling	Definition of Missing	User-defined missing values are treated as missing.
	Cases Used	Statistics for each table are based on all the cases with valid data in the specified range(s) for all variables in each table.
Syntax		CROSSTABS /TABLES=Sexo BY ITUmes3 /FORMAT=AVALUE TABLES /STATISTICS=CHISQ /CELLS=COUNT ROW /COUNT ROUND CELL.
Resources	Processor Time	00:00:00,13
	Elapsed Time	00:00:00,16
	Dimensions Requested	2
	Cells Available	349496

Case Processing Summary

	Cases					
	Valid		Missing		Total	
	N	Percent	N	Percent	N	Percent
Sexo * ITUmes3	784	100,0%	0	0,0%	784	100,0%

Sexo * ITUmes3 Crosstabulation

			ITUmes3					Total
			0	1	2	3	4	
Sexo	Mujer	Count	294	185	138	28	3	648
		% within Sexo	45,4%	28,5%	21,3%	4,3%	0,5%	100,0%
	Hombre	Count	52	31	41	12	0	136
		% within Sexo	38,2%	22,8%	30,1%	8,8%	0,0%	100,0%
Total		Count	346	216	179	40	3	784
		% within Sexo	44,1%	27,6%	22,8%	5,1%	0,4%	100,0%

Chi-Square Tests

	Value	df	Asymptotic Significance (2-sided)
Pearson Chi-Square	11,601[a]	4	,021
Likelihood Ratio	11,328	4	,023
Linear-by-Linear Association	6,962	1	,008
N of Valid Cases	784		

a. 2 cells (20,0%) have expected count less than 5. The minimum expected count is ,52.

```
CROSSTABS
 /TABLES=Sexo BY ITUmes6
 /FORMAT=AVALUE TABLES
 /STATISTICS=CHISQ
 /CELLS=COUNT ROW
 /COUNT ROUND CELL.
```

Crosstabs

Notes

Output Created		14-SEP-2019 23:15:31
Input	Data	C: \Users\mm\Documents\CONGRESOS 2017 Treballs\EUROPEO LONDON 2017 VACUNAS UROMUNE\VACUNAS_Base Dades_BMC.sav
	Active Dataset	DataSet2
	Filter	<none>
	Weight	<none>
	Split File	<none>
	N of Rows in Working Data File	784
Missing Value Handling	Definition of Missing	User-defined missing values are treated as missing.
	Cases Used	Statistics for each table are based on all the cases with valid data in the specified range(s) for all variables in each table.
Syntax		CROSSTABS /TABLES=Sexo BY ITUmes6 /FORMAT=AVALUE TABLES /STATISTICS=CHISQ /CELLS=COUNT ROW /COUNT ROUND CELL.
Resources	Processor Time	00:00:00,05
	Elapsed Time	00:00:00,12
	Dimensions Requested	2
	Cells Available	349496

Case Processing Summary

	Cases					
	Valid		Missing		Total	
	N	Percent	N	Percent	N	Percent
Sexo * ITUmes6	784	100,0%	0	0,0%	784	100,0%

Sexo * ITUmes6 Crosstabulation

			ITUmes6					
			0	1	2	3	4	5
Sexo	Mujer	Count	212	215	151	64	3	3
		% within Sexo	32,7%	33,2%	23,3%	9,9%	0,5%	0,5%
	Hombre	Count	41	39	31	22	3	0
		% within Sexo	30,1%	28,7%	22,8%	16,2%	2,2%	0,0%
Total		Count	253	254	182	86	6	3
		% within Sexo	32,3%	32,4%	23,2%	11,0%	0,8%	0,4%

Sexo * ITUmes6 Crosstabulation

			Total
Sexo	Mujer	Count	648
		% within Sexo	100,0%
	Hombre	Count	136
		% within Sexo	100,0%
Total		Count	784
		% within Sexo	100,0%

Chi-Square Tests

	Value	df	Asymptotic Significance (2-sided)
Pearson Chi-Square	10,104[a]	5	,072
Likelihood Ratio	9,110	5	,105
Linear-by-Linear Association	3,333	1	,068
N of Valid Cases	784		

a. 4 cells (33,3%) have expected count less than 5. The minimum expected count is ,52.

```
 DO IF  (Sexo = 0).
RECODE EDAD (50 thru Highest=1) INTO menopausicas.
END IF.
EXECUTE.


FREQUENCIES VARIABLES=menopausicas
  /ORDER=ANALYSIS.
```

Frequencies

Notes

Output Created		14-SEP-2019 23:15:31
Input	Data	C:<br>\Users\mm\Documents\CONGRESOS 2017 Treballs\EUROPEO LONDON 2017 VACUNAS UROMUNE\VACUNAS_Base Dades_BMC.sav
	Active Dataset	DataSet2
	Filter	<none>
	Weight	<none>
	Split File	<none>
	N of Rows in Working Data File	784
Missing Value Handling	Definition of Missing	User-defined missing values are treated as missing.
	Cases Used	Statistics are based on all cases with valid data.
Syntax		FREQUENCIES VARIABLES=menopausicas<br>/ORDER=ANALYSIS.
Resources	Processor Time	00:00:00,02
	Elapsed Time	00:00:00,03

Statistics

menopausicas

N	Valid	613
	Missing	171

menopausicas

		Frequency	Percent	Valid Percent	Cumulative Percent
Valid	1,00	613	78,2	100,0	100,0
Missing	System	171	21,8		
Total		784	100,0		

```
CROSSTABS
 /TABLES=menopausicas BY ITUmes0
 /FORMAT=AVALUE TABLES
 /STATISTICS=CHISQ
 /CELLS=COUNT ROW COLUMN TOTAL
 /COUNT ROUND CELL.
```

Crosstabs

Notes

Output Created		14-SEP-2019 23:15:31
Input	Data	C: \Users\mm\Documents\CONGRESOS 2017 Treballs\EUROPEO LONDON 2017 VACUNAS UROMUNE\VACUNAS_Base Dades_BMC.sav
	Active Dataset	DataSet2
	Filter	<none>
	Weight	<none>
	Split File	<none>
	N of Rows in Working Data File	784
Missing Value Handling	Definition of Missing	User-defined missing values are treated as missing.
	Cases Used	Statistics for each table are based on all the cases with valid data in the specified range(s) for all variables in each table.
Syntax		CROSSTABS /TABLES=menopausicas BY ITUmes0 /FORMAT=AVALUE TABLES /STATISTICS=CHISQ /CELLS=COUNT ROW COLUMN TOTAL /COUNT ROUND CELL.
Resources	Processor Time	00:00:00,05
	Elapsed Time	00:00:00,04
	Dimensions Requested	2
	Cells Available	349496

Case Processing Summary

	Cases					
	Valid		Missing		Total	
	N	Percent	N	Percent	N	Percent
menopausicas * ITUmes0	613	78,2%	171	21,8%	784	100,0%

menopausicas * ITUmes0 Crosstabulation

			ITUmes0			
			3	4	5	6
menopausicas	1,00	Count	225	173	120	59
		% within menopausicas	36,7%	28,2%	19,6%	9,6%
		% within ITUmes0	100,0%	100,0%	100,0%	100,0%
		% of Total	36,7%	28,2%	19,6%	9,6%
Total		Count	225	173	120	59
		% within menopausicas	36,7%	28,2%	19,6%	9,6%
		% within ITUmes0	100,0%	100,0%	100,0%	100,0%
		% of Total	36,7%	28,2%	19,6%	9,6%

menopausicas * ITUmes0 Crosstabulation

			ITUmes0			
			7	8	9	10
menopausicas	1,00	Count	24	10	1	1
		% within menopausicas	3,9%	1,6%	0,2%	0,2%
		% within ITUmes0	100,0%	100,0%	100,0%	100,0%
		% of Total	3,9%	1,6%	0,2%	0,2%
Total		Count	24	10	1	1
		% within menopausicas	3,9%	1,6%	0,2%	0,2%
		% within ITUmes0	100,0%	100,0%	100,0%	100,0%
		% of Total	3,9%	1,6%	0,2%	0,2%

menopausicas * ITUmes0 Crosstabulation

			Total
menopausicas	1,00	Count	613
		% within menopausicas	100,0%
		% within ITUmes0	100,0%
		% of Total	100,0%
Total		Count	613
		% within menopausicas	100,0%
		% within ITUmes0	100,0%
		% of Total	100,0%

Chi-Square Tests

	Value
Pearson Chi-Square	.[a]
N of Valid Cases	613

a. No statistics are computed because menopausicas is a constant.

```
CROSSTABS
  /TABLES=menopausicas BY ITUmes3
  /FORMAT=AVALUE TABLES
  /STATISTICS=CHISQ
  /CELLS=COUNT ROW COLUMN TOTAL
```

Crosstabs

Notes

Output Created		14-SEP-2019 23:15:31
Input	Data	C: \Users\mm\Documents\CONGRESOS 2017 Treballs\EUROPEO LONDON 2017 VACUNAS UROMUNE\VACUNAS_Base Dades_BMC.sav
	Active Dataset	DataSet2
	Filter	<none>
	Weight	<none>
	Split File	<none>
	N of Rows in Working Data File	784
Missing Value Handling	Definition of Missing	User-defined missing values are treated as missing.
	Cases Used	Statistics for each table are based on all the cases with valid data in the specified range(s) for all variables in each table.
Syntax		CROSSTABS /TABLES=menopausicas BY ITUmes3 /FORMAT=AVALUE TABLES /STATISTICS=CHISQ /CELLS=COUNT ROW COLUMN TOTAL /COUNT ROUND CELL.
Resources	Processor Time	00:00:00,05
	Elapsed Time	00:00:00,05
	Dimensions Requested	2
	Cells Available	349496

Case Processing Summary

	Cases					
	Valid		Missing		Total	
	N	Percent	N	Percent	N	Percent
menopausicas * ITUmes3	613	78,2%	171	21,8%	784	100,0%

menopausicas * ITUmes3 Crosstabulation

			ITUmes3			
			0	1	2	3
menopausicas	1,00	Count	286	171	127	26
		% within menopausicas	46,7%	27,9%	20,7%	4,2%
		% within ITUmes3	100,0%	100,0%	100,0%	100,0%
		% of Total	46,7%	27,9%	20,7%	4,2%
Total		Count	286	171	127	26
		% within menopausicas	46,7%	27,9%	20,7%	4,2%
		% within ITUmes3	100,0%	100,0%	100,0%	100,0%
		% of Total	46,7%	27,9%	20,7%	4,2%

menopausicas * ITUmes3 Crosstabulation

			ITUmes3	
			4	Total
menopausicas	1,00	Count	3	613
		% within menopausicas	0,5%	100,0%
		% within ITUmes3	100,0%	100,0%
		% of Total	0,5%	100,0%
Total		Count	3	613
		% within menopausicas	0,5%	100,0%
		% within ITUmes3	100,0%	100,0%
		% of Total	0,5%	100,0%

Chi-Square Tests

	Value
Pearson Chi-Square	.[a]
N of Valid Cases	613

a. No statistics are computed because menopausicas is a constant.

```
CROSSTABS
 /TABLES=menopausicas BY ITUmes6
```

```
/FORMAT=AVALUE TABLES
/STATISTICS=CHISQ
/CELLS=COUNT ROW COLUMN TOTAL
/COUNT ROUND CELL.
```

Crosstabs

Notes

Output Created		14-SEP-2019 23:15:31
Input	Data	C:<br>\Users\mm\Documents\CONGRESOS 2017 Treballs\EUROPEO LONDON 2017 VACUNAS UROMUNE\VACUNAS_Base Dades_BMC.sav
	Active Dataset	DataSet2
	Filter	<none>
	Weight	<none>
	Split File	<none>
	N of Rows in Working Data File	784
Missing Value Handling	Definition of Missing	User-defined missing values are treated as missing.
	Cases Used	Statistics for each table are based on all the cases with valid data in the specified range(s) for all variables in each table.
Syntax		CROSSTABS<br>/TABLES=menopausicas BY ITUmes6<br>/FORMAT=AVALUE TABLES<br>/STATISTICS=CHISQ<br>/CELLS=COUNT ROW COLUMN TOTAL<br>/COUNT ROUND CELL.
Resources	Processor Time	00:00:00,08
	Elapsed Time	00:00:00,09
	Dimensions Requested	2
	Cells Available	349496

Case Processing Summary

	Cases					
	Valid		Missing		Total	
	N	Percent	N	Percent	N	Percent
menopausicas * ITUmes6	613	78,2%	171	21,8%	784	100,0%

menopausicas * ITUmes6 Crosstabulation

			ITUmes6			
			0	1	2	3
menopausicas	1,00	Count	207	201	141	60
		% within menopausicas	33,8%	32,8%	23,0%	9,8%
		% within ITUmes6	100,0%	100,0%	100,0%	100,0%
		% of Total	33,8%	32,8%	23,0%	9,8%
Total		Count	207	201	141	60
		% within menopausicas	33,8%	32,8%	23,0%	9,8%
		% within ITUmes6	100,0%	100,0%	100,0%	100,0%
		% of Total	33,8%	32,8%	23,0%	9,8%

menopausicas * ITUmes6 Crosstabulation

			ITUmes6		Total
			4	5	
menopausicas	1,00	Count	1	3	613
		% within menopausicas	0,2%	0,5%	100,0%
		% within ITUmes6	100,0%	100,0%	100,0%
		% of Total	0,2%	0,5%	100,0%
Total		Count	1	3	613
		% within menopausicas	0,2%	0,5%	100,0%
		% within ITUmes6	100,0%	100,0%	100,0%
		% of Total	0,2%	0,5%	100,0%

Chi-Square Tests

	Value
Pearson Chi-Square	.[a]
N of Valid Cases	613

a. No statistics are computed because menopausicas is a constant.

```
 DO IF  (Sexo = 0).
RECODE EDAD (0 thru 50=1) INTO NOmenopausicas.
END IF.
EXECUTE.
```

```
FREQUENCIES VARIABLES=NOmenopausicas
  /ORDER=ANALYSIS.
```

Frequencies

Notes

Output Created		14-SEP-2019 23:15:32
Input	Data	C:\Users\mm\Documents\CONGRESOS 2017 Treballs\EUROPEO LONDON 2017 VACUNAS UROMUNE\VACUNAS_Base Dades_BMC.sav
	Active Dataset	DataSet2
	Filter	<none>
	Weight	<none>
	Split File	<none>
	N of Rows in Working Data File	784
Missing Value Handling	Definition of Missing	User-defined missing values are treated as missing.
	Cases Used	Statistics are based on all cases with valid data.
Syntax		FREQUENCIES VARIABLES=NOmenopausicas /ORDER=ANALYSIS.
Resources	Processor Time	00:00:00,02
	Elapsed Time	00:00:00,01

Statistics

NOmenopausicas

N	Valid	648
	Missing	136

NOmenopausicas

		Frequency	Percent	Valid Percent	Cumulative Percent
Valid	,00	600	76,5	92,6	92,6
	1,00	48	6,1	7,4	100,0
	Total	648	82,7	100,0	
Missing	System	136	17,3		
Total		784	100,0		

```
 DO IF  (Sexo = 0).
RECODE EDAD (0 thru 50=1) (50 thru Highest=0) INTO NOmenopausicas.
END IF.
EXECUTE.


CROSSTABS
```

```
/TABLES=NOmenopausicas BY ITUmes0
/FORMAT=AVALUE TABLES
/STATISTICS=CHISQ
/CELLS=COUNT ROW COLUMN TOTAL
/COUNT ROUND CELL.
```

Crosstabs

Notes

Output Created		14-SEP-2019 23:15:32
Input	Data	C:\Users\mm\Documents\CONGRESOS 2017 Treballs\EUROPEO LONDON 2017 VACUNAS UROMUNE\VACUNAS_Base Dades_BMC.sav
	Active Dataset	DataSet2
	Filter	<none>
	Weight	<none>
	Split File	<none>
	N of Rows in Working Data File	784
Missing Value Handling	Definition of Missing	User-defined missing values are treated as missing.
	Cases Used	Statistics for each table are based on all the cases with valid data in the specified range(s) for all variables in each table.
Syntax		CROSSTABS /TABLES=NOmenopausicas BY ITUmes0 /FORMAT=AVALUE TABLES /STATISTICS=CHISQ /CELLS=COUNT ROW COLUMN TOTAL /COUNT ROUND CELL.
Resources	Processor Time	00:00:00,03
	Elapsed Time	00:00:00,03
	Dimensions Requested	2
	Cells Available	349496

Case Processing Summary

	Cases					
	Valid		Missing		Total	
	N	Percent	N	Percent	N	Percent
NOmenopausicas * ITUmes0	648	82,7%	136	17,3%	784	100,0%

NOmenopausicas * ITUmes0 Crosstabulation

			ITUmes0			
			3	4	5	6
NOmenopausicas	,00	Count	224	172	120	59
		% within NOmenopausicas	36,7%	28,2%	19,6%	9,7%
		% within ITUmes0	92,9%	94,5%	95,2%	95,2%
		% of Total	34,6%	26,5%	18,5%	9,1%
	1,00	Count	17	10	6	3
		% within NOmenopausicas	45,9%	27,0%	16,2%	8,1%
		% within ITUmes0	7,1%	5,5%	4,8%	4,8%
		% of Total	2,6%	1,5%	0,9%	0,5%
Total		Count	241	182	126	62
		% within NOmenopausicas	37,2%	28,1%	19,4%	9,6%
		% within ITUmes0	100,0%	100,0%	100,0%	100,0%
		% of Total	37,2%	28,1%	19,4%	9,6%

NOmenopausicas * ITUmes0 Crosstabulation

			ITUmes0			
			7	8	9	10
NOmenopausicas	,00	Count	24	10	1	1
		% within NOmenopausicas	3,9%	1,6%	0,2%	0,2%
		% within ITUmes0	96,0%	100,0%	100,0%	100,0%
		% of Total	3,7%	1,5%	0,2%	0,2%
	1,00	Count	1	0	0	0
		% within NOmenopausicas	2,7%	0,0%	0,0%	0,0%
		% within ITUmes0	4,0%	0,0%	0,0%	0,0%
		% of Total	0,2%	0,0%	0,0%	0,0%
Total		Count	25	10	1	1
		% within NOmenopausicas	3,9%	1,5%	0,2%	0,2%
		% within ITUmes0	100,0%	100,0%	100,0%	100,0%
		% of Total	3,9%	1,5%	0,2%	0,2%

NOmenopausicas * ITUmes0 Crosstabulation

			Total
NOmenopausicas	,00	Count	611
		% within NOmenopausicas	100,0%
		% within ITUmes0	94,3%
		% of Total	94,3%
	1,00	Count	37
		% within NOmenopausicas	100,0%
		% within ITUmes0	5,7%
		% of Total	5,7%
Total		Count	648
		% within NOmenopausicas	100,0%
		% within ITUmes0	100,0%
		% of Total	100,0%

Chi-Square Tests

	Value	df	Asymptotic Significance (2-sided)
Pearson Chi-Square	1,984[a]	7	,961
Likelihood Ratio	2,647	7	,916
Linear-by-Linear Association	1,692	1	,193
N of Valid Cases	648		

a. 7 cells (43,8%) have expected count less than 5. The minimum expected count is ,06.

```
CROSSTABS
 /TABLES=NOmenopausicas BY ITUmes3
 /FORMAT=AVALUE TABLES
 /STATISTICS=CHISQ
 /CELLS=COUNT ROW COLUMN TOTAL
 /COUNT ROUND CELL.
```

Crosstabs

Notes

Output Created		14-SEP-2019 23:15:32
Input	Data	C: \Users\mm\Documents\CONGRESOS 2017 Treballs\EUROPEO LONDON 2017 VACUNAS UROMUNE\VACUNAS_Base Dades_BMC.sav
	Active Dataset	DataSet2
	Filter	<none>
	Weight	<none>
	Split File	<none>
	N of Rows in Working Data File	784
Missing Value Handling	Definition of Missing	User-defined missing values are treated as missing.
	Cases Used	Statistics for each table are based on all the cases with valid data in the specified range(s) for all variables in each table.
Syntax		CROSSTABS<br>/TABLES=NOmenopausicas BY ITUmes3<br>/FORMAT=AVALUE TABLES<br>/STATISTICS=CHISQ<br>/CELLS=COUNT ROW COLUMN TOTAL<br>/COUNT ROUND CELL.
Resources	Processor Time	00:00:00,03
	Elapsed Time	00:00:00,04
	Dimensions Requested	2
	Cells Available	349496

Case Processing Summary

	Cases					
	Valid		Missing		Total	
	N	Percent	N	Percent	N	Percent
NOmenopausicas * ITUmes3	648	82,7%	136	17,3%	784	100,0%

NOmenopausicas * ITUmes3 Crosstabulation

			ITUmes3			
			0	1	2	3
NOmenopausicas	,00	Count	284	171	127	26
		% within NOmenopausicas	46,5%	28,0%	20,8%	4,3%
		% within ITUmes3	96,6%	92,4%	92,0%	92,9%
		% of Total	43,8%	26,4%	19,6%	4,0%
	1,00	Count	10	14	11	2
		% within NOmenopausicas	27,0%	37,8%	29,7%	5,4%
		% within ITUmes3	3,4%	7,6%	8,0%	7,1%
		% of Total	1,5%	2,2%	1,7%	0,3%
Total		Count	294	185	138	28
		% within NOmenopausicas	45,4%	28,5%	21,3%	4,3%
		% within ITUmes3	100,0%	100,0%	100,0%	100,0%
		% of Total	45,4%	28,5%	21,3%	4,3%

NOmenopausicas * ITUmes3 Crosstabulation

			ITUmes3	
			4	Total
NOmenopausicas	,00	Count	3	611
		% within NOmenopausicas	0,5%	100,0%
		% within ITUmes3	100,0%	94,3%
		% of Total	0,5%	94,3%
	1,00	Count	0	37
		% within NOmenopausicas	0,0%	100,0%
		% within ITUmes3	0,0%	5,7%
		% of Total	0,0%	5,7%
Total		Count	3	648
		% within NOmenopausicas	0,5%	100,0%
		% within ITUmes3	100,0%	100,0%
		% of Total	0,5%	100,0%

Chi-Square Tests

	Value	df	Asymptotic Significance (2-sided)
Pearson Chi-Square	5,695[a]	4	,223
Likelihood Ratio	6,086	4	,193
Linear-by-Linear Association	3,454	1	,063
N of Valid Cases	648		

a. 3 cells (30,0%) have expected count less than 5. The minimum expected count is ,17.

```
CROSSTABS
  /TABLES=NOmenopausicas BY ITUmes6
  /FORMAT=AVALUE TABLES
  /STATISTICS=CHISQ
  /CELLS=COUNT ROW COLUMN TOTAL
  /COUNT ROUND CELL.
```

Crosstabs

Notes

Output Created		14-SEP-2019 23:15:32
Input	Data	C: \Users\mm\Documents\CONGRESOS 2017 Treballs\EUROPEO LONDON 2017 VACUNAS UROMUNE\VACUNAS_Base Dades_BMC.sav
	Active Dataset	DataSet2
	Filter	<none>
	Weight	<none>
	Split File	<none>
	N of Rows in Working Data File	784
Missing Value Handling	Definition of Missing	User-defined missing values are treated as missing.
	Cases Used	Statistics for each table are based on all the cases with valid data in the specified range(s) for all variables in each table.
Syntax		CROSSTABS /TABLES=NOmenopausicas BY ITUmes6 /FORMAT=AVALUE TABLES /STATISTICS=CHISQ /CELLS=COUNT ROW COLUMN TOTAL /COUNT ROUND CELL.
Resources	Processor Time	00:00:00,02
	Elapsed Time	00:00:00,03
	Dimensions Requested	2
	Cells Available	349496

Case Processing Summary

	Cases					
	Valid		Missing		Total	
	N	Percent	N	Percent	N	Percent
NOmenopausicas * ITUmes6	648	82,7%	136	17,3%	784	100,0%

NOmenopausicas * ITUmes6 Crosstabulation

			ITUmes6			
			0	1	2	3
NOmenopausicas	,00	Count	205	201	141	60
		% within NOmenopausicas	33,6%	32,9%	23,1%	9,8%
		% within ITUmes6	96,7%	93,5%	93,4%	93,8%
		% of Total	31,6%	31,0%	21,8%	9,3%
	1,00	Count	7	14	10	4
		% within NOmenopausicas	18,9%	37,8%	27,0%	10,8%
		% within ITUmes6	3,3%	6,5%	6,6%	6,3%
		% of Total	1,1%	2,2%	1,5%	0,6%
Total		Count	212	215	151	64
		% within NOmenopausicas	32,7%	33,2%	23,3%	9,9%
		% within ITUmes6	100,0%	100,0%	100,0%	100,0%
		% of Total	32,7%	33,2%	23,3%	9,9%

NOmenopausicas * ITUmes6 Crosstabulation

			ITUmes6		Total
			4	5	
NOmenopausicas	,00	Count	1	3	611
		% within NOmenopausicas	0,2%	0,5%	100,0%
		% within ITUmes6	33,3%	100,0%	94,3%
		% of Total	0,2%	0,5%	94,3%
	1,00	Count	2	0	37
		% within NOmenopausicas	5,4%	0,0%	100,0%
		% within ITUmes6	66,7%	0,0%	5,7%
		% of Total	0,3%	0,0%	5,7%
Total		Count	3	3	648
		% within NOmenopausicas	0,5%	0,5%	100,0%
		% within ITUmes6	100,0%	100,0%	100,0%
		% of Total	0,5%	0,5%	100,0%

Chi-Square Tests

	Value	df	Asymptotic Significance (2-sided)
Pearson Chi-Square	23,695[a]	5	,000
Likelihood Ratio	11,277	5	,046
Linear-by-Linear Association	3,899	1	,048
N of Valid Cases	648		

a. 5 cells (41,7%) have expected count less than 5. The minimum expected count is ,17.

```
DATASET ACTIVATE DataSet2.

SAVE OUTFILE='C:\Users\mm\Documents\CONGRESOS 2017 Treballs\EUROPEO LONDON 2017 VACUNAS '+
    'UROMUNE\VACUNAS_Base Dades_BMC.sav'
  /COMPRESSED.
DATASET ACTIVATE DataSet1.
DATASET CLOSE DataSet2.
```
